# A case report of a giant appendiceal mucocele and literature review

**DOI:** 10.11604/pamj.2017.28.106.13832

**Published:** 2017-10-04

**Authors:** Mpapho Joseph Motsumi, Pako Motlaleselelo, Gezahen Ayane, Sheikh Omar Sesay, Johamel Ramos Valdes

**Affiliations:** 1 University of Botswana, Department of Surgery, Sir Ketumile Masire University Hospital, PO Box, Mogoditshane, Botswana; 2 Department of Surgery, Ministry of Health, Princess Marina Hospital, Mogoditshane, Botswana; 3 Department of Surgery, University of Botswana, Sir Ketumile Masire University Hospital, Mogoditshane, Botswana; 4 Ministry of Health, Department of Radiol, Princess Marina Hospital, Mogoditshane, Botswana; 5 Department of Pathology, University of Botswana, Sir Ketumile Masire University Hospital, Mogoditshane, Botswana

**Keywords:** Appendicular mucocele, appendicular mucinous cystadenoma, appendicular mucinous cystadenocarcinoma, appendicular mucinous adenocarcinoma, pseudomyxoma peritonei, low-grade appendiceal mucinous neoplasms, mucinous neoplasms of the appendix

## Abstract

A 43-year-old female presented at the accident and emergency department of Princess Marina Hospital, Gaborone, Botswana. She reported a deep dull aching pain of two years duration in the right iliac fossa that has been progressively becoming worse. Ultrasound revealed a large sausage like cystic mass extending from the pelvis up to the medial aspect of the ascending colon. CT scan showed a large sausage like cystic mass extending from the pelvis up to the hepatic flexure of the colon with the cecum displaced. No metastatic features were seen. We made an impression of appendiceal mucocele. A semi-elective laparotomy was scheduled. Intraoperative findings: a giant intact cystic distended appendix with involved base, displacing the cecum cranially. A right hemicolectomy was performed. The histopathological results revealed a low-grade appendicular mucinous neoplasm with no lymph node involvement. The surgical margins were free. The patient recovered uneventfully.

## Introduction

Rokitansky first described Appendiceal mucocele in 1842. It is a mucinous distention of the appendix, which can be of neoplastic or non-neoplastic origin [1, 2]. It is uncommon and has an incidence of 0.2%-0.3% of all appendectomies performed and 8%-10% of all appendiceal tumors [3, 4]. It can be asymptomatic and often diagnosed incidentally or may present with appendicitis-like symptoms [3, 5, 6]. It rarely presents as intestinal obstruction however, such cases have been reported [5]. Four types of appendiceal mucocele are defined by the cause of obstruction: retention cysts, epithelial hyperplasia, mucinous cystadenoma and mucinous cystadenocarcinoma [7]. Appendiceal mucocele can be either benign or malignant. Low‐grade appendiceal mucinous neoplasms (LAMNs) is used to refer to mucinous cystadenomas. Mucinous tumors account for 58% of malignant tumors of appendix in SEER database and the remaining are carcinoids [8]. A preoperative diagnosis is crucial in order to choose the correct operative management. The correct surgical management depends on size and location of lesion. Appendiceal mucocele can be difficult to differentiate from an adnexal mass even on good imaging [9]. Abdominal CT scan is a better diagnostic tool [10]. Spontaneous or iatrogenic rupture of mucinous appendiceal tumors lead to spread to the peritoneum and viscera as a gelatinous material resulting in a condition known as pseudomyxoma peritonei. Pseudomyxoma peritonei is associated with long-term morbidity and mortality. The ultimate goal of management is to avoid rupture of appendiceal mucocele and the syndrome of pseudomyxoma peritonei. Laparotomy is the traditionally recommended approach, even though case reports of minimally invasive surgical approaches are increasingly being reported as safe [10]. A decision to perform a right hemicolectomy should be influenced by the criteria reported by Gonzalez-Moreno [10]. Surgical resection is the only potentially curative approach. Accepted management includes appendectomy, right hemicolectomy, partial colectomy with debulking or palliative resection combined with additional chemotherapy [11]. Cytoreductive surgery (CRS) and hyperthermic intraperitoneal chemotherapy (HIPEC) provides long-term survival in selected patients with these conditions [12]. The use of the combined modality treatment of cytoreductive surgery (CRS) and hyperthermic intraperitoneal chemotherapy (HIPEC) has led to a 5-year survival ranging from 62.5% to 100% for low grade and 0%-65% for high-grade disease [7].

## Patient and observation

**Figure 1:**
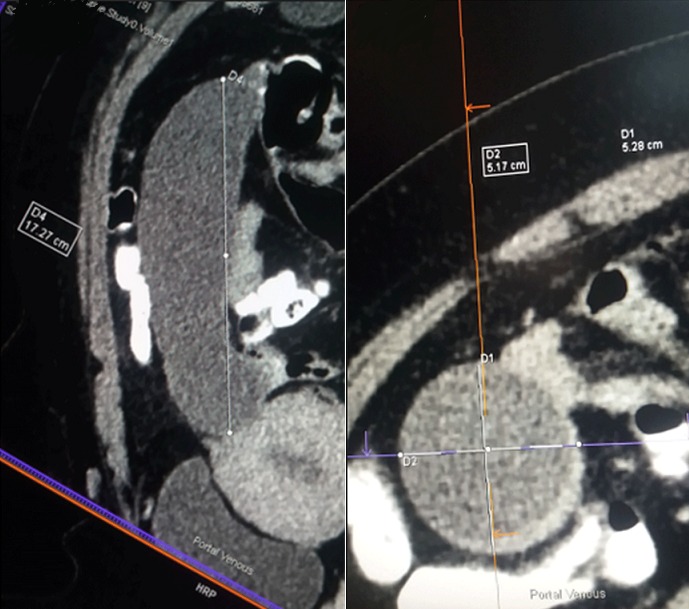
CT-scan images

**Figure 2:**
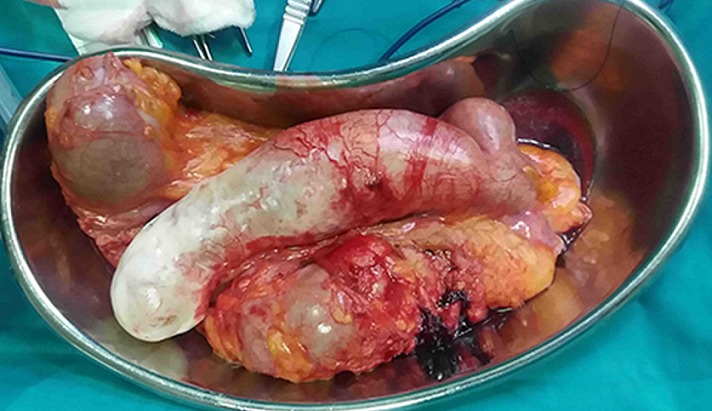
Surgical specimen

We present a case of a 43-year-old female who presented at the accident and emergency department of Princess Marina Hospital, Gaborone, Botswana. She was referred from a peripheral hospital. She reported a deep dull aching pain in the right iliac fossa that has been progressively becoming worse. She had no associated vomiting or change in bowel habits. She reported no weight loss. The pain was of two years duration and has been in and out of hospital on antibiotics and analgesia. She was referred to Princess Marina Hospital after having been found to have a large sausage like cystic mass extending from the pelvis up to the medial aspect of the ascending colon on ultrasound. Its origins were unclear. There was no free fluid seen on ultrasound. The patient was otherwise afebrile and hemodynamically stable. She had normal full blood count, urea and electrolytes, liver function tests and pregnancy was negative. She was admitted to the ward on analgesia and an abdomino-pelvic CT scan was performed the next day, which showed a sausage like cystic mass extending from the pelvis up to the hepatic flexure of the colon with the cecum displaced and ante-flexed over the ascending colon. There were no liver lesions or lymphadenopathy noted. The length of the mass was 17.27 cm and the maximum diameter was 5.28cm (Figure 1). There was no free fluid in the abdomen. An impression of appendiceal mucocele was made after the surgeon and the radiologist carefully analysed the CT scan. A semi-elective right hemi-colectomy was planned. Intraoperative findings (Figure 2): a distended massive appendix pushing the redundant cecal end up to the hepatic flexure of the colon and the tip of the appendix into the right iliac fossa was found. Exploration of the abdomen revealed no metastatic processes, no lymphadenopathy and no other malignancies. The pelvic organs were all normal. There was no leak of appendicular contents and the appendix was not adherent to surrounding structures. The distended appendix was isolated carefully and delivered from the abdomen. A right hemicolectomy was performed making sure not to rupture the appendix or cause any leakage of its contents. A stapled functional end-to-end ileo-colic anastomosis was performed. The patient recovery was uneventful and was discharged day 6 post-operation. She was seen at the outpatient clinic 6 weeks later and had uneventful postoperative course. Histology results revealed a low-grade appendicular mucinous neoplasm with negative margins and no involved mesenteric lymph nodes.

### Histopathology report

### *All slide sections are stained with Haematoxylin & Eosin (H/E)

**Figure 3:**
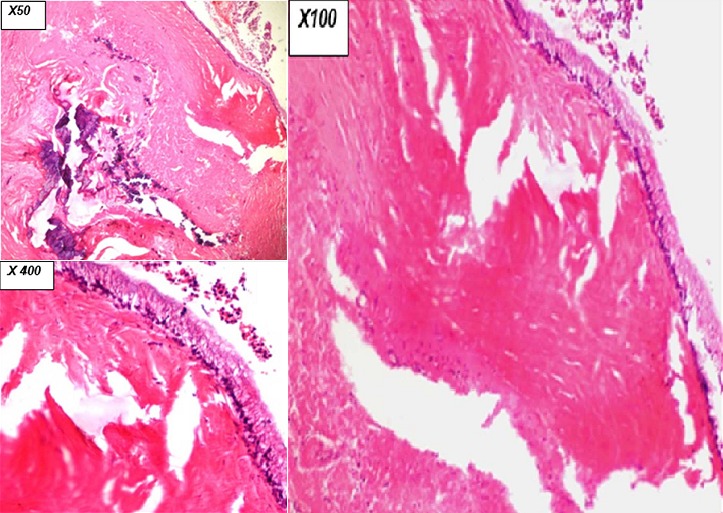
Histopathology slide x50, x100, x400

**X50 (**Figure 3**): low power viewing)**: The appendiceal wall shows a flattened and misplaced epithelium (on top) as well as marked fibrosis surrounding a focus of dystrophic calcification (blue stained material in the centre of the image)

**X100 and x400 (**Figure 3)**: medium and high power viewing)**: The appendiceal wall shows an intestinal type covering epithelium (right side) comprising of crowded columnar cells with basal, hyperchromatic nuclei and large amounts of apical mucin as well as minimal dysplastic changes. Marked fibrosis beneath the epithelial lining is readily apparent. *Fibrosis and chronic inflammation are secondary changes due to luminal compression in a long-standing disease.

## Discussion

Preoperative diagnosis of appendiceal mucocele is very important. Contrast-enhanced CT imaging is most commonly used modality for preoperative diagnosis. It informs the choice of procedure and avoids complications. Preoperative colonoscopy may reveal a pathognomonic volcano sign that describes a mass obstructing the appendiceal opening with a central crater that produces mucin [13]. However, the literature is not clear about the role of preoperative colonoscopy in the diagnosis of appendiceal mucocele. Appendiceal mucocele can be benign or malignant and WHO classifies them into four histological types: simple/retention mucocele/retention cyst, which exhibit normal epithelium and mild dilation due to obstruction of appendiceal outflow, often by a fecalith. Hyperplastic epithelium, which exhibit mild luminal distension. Mucinous cystadenoma, also known as low-grade appendiceal mucinous neoplasms (LAMNs) exhibit presence of epithelial atypia and moderate distension. Mucinous cystadenocarcinoma, demonstrating invasion into the appendiceal wall, in addition to features of LAMNs [14]. Mucinous cystadenoma are the most common of the four types. Size is an important factor to consider when dealing with appendiceal mucocele. An appendiceal mucocele that is less than 2cm is rarely malignant and those greater than 6cm are more often associated with cystadenoma and cystadenocarcinoma and a higher rate of perforation [14]. Rupture of either benign or malignant types is associated with pseudomyxoma peritonei, which is associated with a higher morbidity and mortality [15]. Whether the ruptured appendiceal mucocele is benign or malignant has serious prognostic implications. Rupture of benign appendiceal mucocele has a 91-100% 5-year survival rate, while malignant forms have a 5-year survival rate of 25% [16]. Pseudomyxoma peritonei is a difficult condition to manage. Complex surgical interventions may be necessary some of which are aggressive. These interventions include extirpation of mucinous material, debulking, peritonectomy and heated intraperitoneal chemotherapy (HIPEC) [15]. Historically open surgery was recommended as opposed to laparoscopic approach for resection. However, there is ongoing debate in the literature comparing the advantages and disadvantages of open versus laparoscopic approaches. Careful consideration should be given to minimize rupture of the appendiceal mucocele when making a decision on approach of choice. Recent evidence suggests that appendectomy-only is curative for benign, grossly intact mucoceles [17]. González-Moreno and Sugarbaker recommended the use of a sentinel lymph node approach to determine whether a right hemicolectomy is appropriate. Frozen section examination of sentinel lymph nodes within the appendiceal mesentery found along the appendiceal artery are carried out. In the absence of metastatic disease to the lymph nodes, a right hemicolectomy is not indicated [18]. Lymph node metastases secondary to appendiceal mucinous neoplasms rarely occur, occurring in only 4.2% of patients with a mucinous malignancy [19]. Management and algorithms of perforated versus non-perforated mucoceles, positive versus negative mesoappendiceal and ileocolic lymph nodes have been established through the work of Dhage-Ivatury and Sugarbaker. It is recommended that these patients be followed-up clinically and by CT scan for a minimum of 5-10 years post-operatively. Trends of tumor makers (CEA and CA 19-9) may be used with elevation suggesting recurrences [20].

## Conclusion

Appendiceal mucocele is a rare condition. The clinical presentation is often non-specific and the clinician should have appendiceal mucocele in mind in patients presenting with long-term right lower quadrant pain, adnexal masses and acute appendicitis picture. Often, the diagnosis is made incidentally during imaging or surgical procedure. Radiological imaging and careful analysis is critical as it informs choice of procedure and management. Surgical resection is potentially curative and rupture of the mucocele should be avoided as it may lead to pseudomyxoma peritonei, a condition with high morbidity and mortality. Exercise diligence when choosing surgical approach and procedure.

## Competing interests

The authors declare no competing interests.
